# Engineered endothelial cells targeting and dihydrotanshinone I loaded bacterial extracellular vesicles for atherosclerosis therapy

**DOI:** 10.1002/btm2.70074

**Published:** 2025-09-15

**Authors:** Rong‐Rong Zhu, Xue‐Liang Zhou, Yan‐Wei Liu, Ri Xu, Peng Deng, Zhong‐Yong Liu

**Affiliations:** ^1^ Department of Cardiology The Affiliated Hospital of Jiangxi University of Chinese Medicine Nanchang China; ^2^ Department of Cardiovascular Surgery, the First Affiliated Hospital, Jiangxi Medical College Nanchang University Nanchang China

**Keywords:** atherosclerosis, bacterial extracellular vesicles, dihydrotanshinone I, endothelial cells

## Abstract

Atherosclerosis (AS) is a complex cardiovascular disease characterized by endothelial dysfunction, dyslipidemia, and immune‐inflammatory responses, leading to arterial plaque formation and potentially fatal complications such as myocardial infarction and stroke. Traditional treatments, such as statins, often pose challenges due to their side effects and limited efficacy. In this study, we explore a novel therapeutic approach utilizing engineered endothelial cells (ECs) targeting probiotic extracellular vesicles loaded with dihydrotanshinone I (DHT) (EC‐BEVs^DHT^), a bioactive compound derived from Danshen (*Salvia miltiorrhiza* Bunge). With the characterization of EC‐BEVs^DHT^ by transmission electron microscope and nanoparticle tracking analysis, EC‐BEVs^DHT^ exhibited typical spherical morphology and particle size distribution. High‐performance liquid chromatography coupled with tandem mass spectrometric confirmed the expression of the ECs‐targeting peptide VSSSTPR in EC‐BEVs^DHT^ and EC‐BEVs^DHT^. We further investigated the anti‐atherosclerotic effects and molecular mechanisms of EC‐BEVs^DHT^ on human umbilical vein endothelial cells (HUVECs) and Apolipoprotein E‐deficient (ApoE^−/−^) C57BL/6J mice. We found that EC‐BEVs^DHT^ attenuated oxidized low‐density lipoprotein induced HUVECs injury in vitro and decreased AS in ApoE^−/−^ mice in vivo. Our findings suggest that EC‐BEVs^DHT^ hold promise as a safe and effective therapeutic strategy for AS, offering potential advantages over traditional treatments.

**Translational Impact Statement**

This study demonstrates that endothelial‐targeted probiotic extracellular vesicles loaded with dihydrotanshinone I (EC‐BEVs^DHT^) significantly reduce atherosclerosis in preclinical models. By combining cell‐specific targeting with a natural anti‐inflammatory compound in a biocompatible bacterial vesicle carrier, this strategy offers a promising, potentially safer, and more effective alternative to conventional statin therapy. EC‐BEVs^DHT^ addresses key limitations of current treatments by enhancing drug delivery to dysfunctional endothelium and leveraging the synergistic benefits of targeted nanotherapy and natural bioactive agents, paving the way for advanced clinical development in cardiovascular disease.

## 1 INTRODUCTION

In atherosclerosis (AS), a condition characterized by endothelial dysfunction, dyslipidemia, and immune‐inflammatory responses, lipids accumulate beneath the vascular intima, forming irregular atheromatous plaques. These plaques, composed of lipids, smooth muscle cells, inflammatory cells, and necrotic cell fragments, obstruct blood vessels. Consequently, individuals are at risk of fatal conditions such as myocardial infarction and stroke.^1^, ^2^ Statins, commonly prescribed for lipid‐lowering therapy in AS, carry toxic side effects such as rhabdomyolysis, hepatic impairment, and gastrointestinal reactions. These adverse effects can make long‐term usage and regular check‐ups challenging for some patients, leading to increased medical expenses.^3^ Recent research indicates that traditional Chinese medicine, with its millennia‐old history and distinctive therapeutic effects and safety profiles, can ameliorate or treat AS. It achieves this by modulating inflammatory responses, inhibiting oxidative stress, improving macrophage metabolism, suppressing vascular smooth muscle proliferation and migration, and inhibiting extracellular matrix degradation.^4^ Hence, it is conceivable that the development of novel targeted drugs for AS, derived from pure natural herbal medicines, could be promising. These drugs would focus on protecting and remodeling endothelial cells (ECs), potentially leading to the creation of safe, long‐lasting, and affordable treatments for AS.

Danshen refers to the dried roots and rhizomes of the plant *Salvia miltiorrhiza* Bunge, a member of the Lamiaceae family. It has a bitter and slightly cold taste, and it influences the heart and liver meridians while acting on the blood aspect. Danshen is known for its functions in promoting blood circulation, dispersing blood stasis, invigorating blood, relieving pain, alleviating restlessness, and resolving abscesses.^5^ It is primarily used for symptoms such as chest pain due to blood stasis, epigastric and hypochondriac pain, and hot painful obstruction. Modern pharmacological studies have confirmed that Danshen contains over 100 active ingredients, including tanshinones, salvianolic acids, volatile oils, polysaccharides, and nitrogen‐containing compounds. Dihydrotanshinone I (DHT) is one of the lipophilic tanshinones found in the cortex of Danshen, characterized by its unique naphthoquinone structure. Previous studies have demonstrated that DHT exerts significant inhibitory effects on inflammation, oxidative stress, and interstitial calcification. For instance, DHT not only inhibits NF‐κB activation and translocation, thereby suppressing M1 macrophage activation and excessive release of pro‐inflammatory factors.^6^ It also promotes the expression of Nrf2 by enhancing PKM2 glutathionylation, activating antioxidant enzymes to eliminate excessive reactive oxygen species (ROS), thereby alleviating myocardial ischemia–reperfusion injury.^7^ Additionally, it inhibits the influx of Ca^2+^ in vascular smooth muscle cells, dilates coronary arteries, and improves myocardial blood flow during ischemia.^8^ Furthermore, DHT inhibits valvular interstitial cell calcification via the SMAD1/5/8/NF‐κB/ERK signaling pathway^9^ and promotes angiogenesis by upregulating SOX11 expression.^10^ Recent studies suggest that DHT reduces lipid levels and inhibits atherosclerotic plaque formation by diminishing ROS formation, inhibiting NF‐κB translocation, and suppressing the internalization of oxidized low‐density lipoprotein (ox‐LDL) and monocyte adhesion.^11^ Moreover, DHT may stabilize vulnerable plaques and reduce plaque vulnerability by inhibiting macrophage necroptosis mediated by RIP3.^12^ However, despite its clinical value, DHT encounters challenges such as its small molecular weight, short half‐life, poor water solubility, and difficulty in maintaining local drug concentration after intravenous administration. Therefore, improving the bioavailability and tissue targeting of DHT is essential to facilitate its translation and widespread adoption in the clinical treatment of AS.

Bacterial extracellular vesicles (BEVs) are spherical structures secreted by bacteria, typically ranging in diameter from 20 to 400 nm and composed of a lipid bilayer.^13^ They contain lipopolysaccharides, peptidoglycans, cell membranes, periplasmic proteins, toxins, nucleic acids, and more. BEVs serve various functions including cellular communication, quorum sensing, horizontal gene transfer, bacterial killing, toxin delivery, polysaccharide hydrolysis, and stress response.^14^, ^15^ Through biotechnological modification or detoxification, BEVs have emerged as promising novel drug delivery vehicles with clinical applications.^15^ Their advantages include: (1) Rapid bacterial growth, convenient genetic modification, and mature high‐density culture techniques; (2) Easy isolation and release of contents upon internalization by receptor cells; (3) Good tolerance and low immunogenicity, avoiding issues such as immune rejection reactions, poor biocompatibility, high costs, and long treatment times associated with traditional cell therapy; (4) Stable payload capacity, widely used in vaccines, adjuvants, drug delivery, and anti‐infective applications.^16^ Su et al., drawing from the principles of synthetic biology, applied customized probiotic extracellular vesicles to the field of bone tissue engineering.^17^, ^18^, ^19^ Initially, they transformed recombinant plasmids into probiotic Escherichia coli, then introduced target genes into BEVs via electroporation, effectively engineering BEVs with osteoblast‐targeting properties and the capacity to stimulate osteogenic differentiation.^18^, ^19^ *Lactobacillus rhamnosus* GG (LGG), discovered in 1985 by Sherwood Gorbach and Barry Goldwin, is a gram‐positive anaerobic acid‐resistant intestinal symbiotic bacterium that produces L‐lactic acid but no spores. BEVs produced by LGG exhibit distinctive traits, including a cell‐free system, nanometer‐scale structure, stable drug‐loading capacity, good biocompatibility, ease of modification, and suitability for large‐scale production, rendering LGG an ideal candidate for engineered BEVs.^17^, ^20^ In this study, we designed and constructed a novel engineered ECs targeting BEVs loaded with DHT (EC‐BEVs^DHT^). The spherical morphology and uniform particle size distribution were characterized with transmission electron microscope (TEM) and nanoparticle tracking analysis (NTA), respectively. The expression of the ECs‐targeting peptide VSSSTPR in EC‐BEVs^DHT^ and the drug release efficiency were verified by high‐performance liquid chromatography coupled with tandem mass spectrometric (HPLC–MS/MS). In vitro analysis, we found that EC‐BEVs^DHT^ significantly attenuated ox‐LDL induced HUVECs injury. In the Apolipoprotein E‐deficient (ApoE^−/−^) C57BL/6J mice, EC‐BEVs^DHT^ decreased AS in ApoE^−/−^ mice. These results suggest that EC‐BEVs^DHT^ hold promise as a safe and effective therapeutic strategy for AS, potentially offering advantages over traditional treatments.

## 2 MATERIALS AND METHODS

### 2.1 Construction and isolation of EC‐BEVs^DHT^ targeting ECs

**FIGURE 1:**
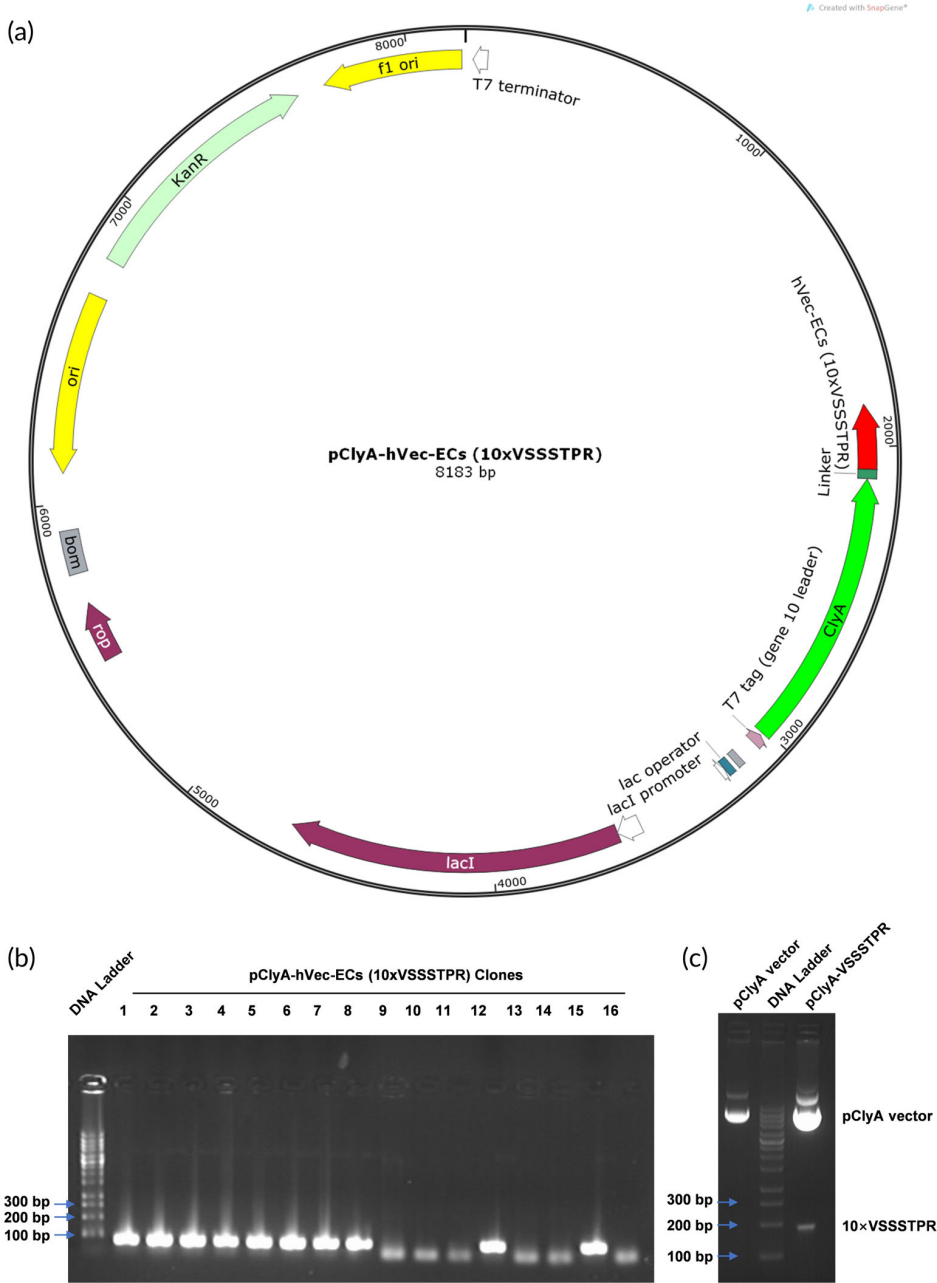
Construction of ECs‐targeting and DHT loaded BEVs. (a) The plasmid map of pClyA‐hVEC‐ECs (10 × VSSSTPR); (b) Positive clone screening of ECs‐targeting peptide expression vector by colony PCR; (c) Validation of ECs‐targeting peptide expression vector by double enzyme digestion.

The preparation of engineered BEVs targeting endothelial cells (EC‐BEVs) involved several steps shown below. Firstly, synthetic biology principles were employed to optimize the codon usage of the human EC‐targeting peptide VSSSTPR for prokaryotic expression in LGG, resulting in the synthesis of the expression element 10xVSSSTPR. Next, a seamless cloning kit was utilized to construct the recombinant plasmid pClyA‐hVec‐ECs (10 × VSSSTPR); the plasmid map was shown in Figure 1a. This recombinant plasmid was then transformed into the probiotic LGG using the calcium chloride (10% CaCl_2_) transformation method, yielding the recombinant strain LGG‐pClyA‐hVec‐ECs. Subsequently, EC‐BEVs were isolated through a series of steps: the fermentation broth was centrifuged at 10,000*g* for 20 min at 4°C to remove bacterial cells; the supernatant was filtered through a 0.22 μm sterile filter (Merck Millipore, USA); the filtrate was concentrated using a 100 kDa ultrafiltration tube (Merck Millipore, USA); the concentrated solution was subjected to ultracentrifugation at 150,000 *g* for 3 h at 4°C with Optiprep density gradient (STEMCELL, USA); and the EVs‐rich fraction was diluted with PBS (Biosharp, Shanghai, China) and subjected to another round of ultracentrifugation at 150,000 *g* for 1–2 h at 4°C to collect EC‐BEVs. To encapsulate dihydrotanshinone I (DHT, MCE, Shanghai, China) into BEVs using electroporation to enhance its therapeutic efficacy. First, prepare a stock solution of DHT in DMSO, ensuring complete dissolution. Dilute the stock solution in PBS to achieve the desired final concentration (100 μg/mL) for loading into BEVs. Combine the resuspended BEVs (50 μg/mL) with the DHT solution (W/W of DHT: BEVs is 2:1) in a sterile electroporation cuvette (Lonza, USA). Set the electroporator to appropriate parameters (250–400 V, 50–100 μF, 3 pulses). After electroporation, immediately transfer the mixture to a recovery medium to stabilize the BEVs. Following electroporation, a portion of the DHT‐loaded EC‐BEVs was collected using the same isolation procedure for subsequent characterization experiments, while the remainder was stored at −80°C for future use. For the characterization of the engineered probiotic and its EC‐BEVs, plasmid double digestion, colony PCR, and agarose gel electrophoresis were used to verify the successful construction of the strain. The morphology, size distribution, and concentration of the EC‐BEVs were observed using TEM and NTA. Additionally, liquid chromatograph‐mass spectrometer (LC–MS, Agilent, USA) was employed to confirm the presence of the human EC‐targeting peptide VSSSTPR within the vesicles.

### 2.2 TEM observation of BEVs morphology

10 μL of BEVs was placed on a copper grid (Sigma Aldrich, USA), allowed to settle for 1 min, and excess liquid was removed with filter paper. Subsequently, 10 μL of uranyl acetate (Sigma Aldrich, USA) was added, and the grid was treated similarly. After air‐drying at room temperature for several minutes, imaging was conducted at 100 kV using an electron microscope (Hitachi, HT‐7700).

### 2.3 NTA particle size detection

NTA particle size was analyzed using a nanoparticle tracking analyzer (ZetaVIEW NTA, PARTICLE METRIX). Briefly, ensure frozen samples are completely thawed at 25°C. Dilute the BEVs sample in 1 × PBS (Biosharp, Shanghai, China) to a concentration of 1 × 10^9^ particles/mL for analysis. Set the minimum number of detectable particles per frame to 5–10 particles/frame to ensure reliable size and concentration calculations. Perform 10 cycles per sample acquisition, allowing for adequate averaging of particle movement for improved accuracy. Acquire data from 3 different positions within each sample chamber to account for any potential heterogeneity in particle distribution. Set the shutter gain to 80%, which balances light intensity without causing saturation. Use a sensitivity setting of 80%–100% to ensure detection of smaller particles while maintaining a good signal‐to‐noise ratio. Adjust the detection threshold to 1–3. Ensure size calibration is performed regularly using standard reference particles to maintain accuracy in size measurements. Save data in the .csv format for further analysis and reporting. Analyze particle size distribution and concentration using built‐in software features.

### 2.4 HPLC–MS/MS detection of DHT content

Use 1 mg of the BEVs preparation containing DHT for extraction. Add 1 mL of methanol to the 1 mg sample. Subject the sample‐methanol mixture to sonication for 30 min at room temperature in a sonication bath. Ensure that the amplitude is set to moderate levels (50% power) to avoid overheating the sample. After sonication, centrifuge the mixture at 14,000 × *g* for 10 min at 4°C to separate the solid debris from the supernatant. Carefully collect the supernatant for further processing. Evaporate the collected supernatant under nitrogen flow at 45°C until dry. Alternatively, use a vacuum concentrator to achieve complete evaporation, which may take approximately 2 h. Reconstitute the dried residue in 200 μL of 5 mM ammonium acetate in water. This media is chosen to maintain compatibility with the mobile phase during LC–MS/MS analysis using a liquid chromatography–tandem mass spectrometry (LC–MS/MS) system (TSQ VANTAGE, Thermo Scientific, USA). Filter the reconstituted solution using a 0.22 μm syringe filter to remove any particulate matter that may interfere with the LC–MS/MS analysis.

### 2.5 Protein quantification of BEVs

A BCA Protein Assay Kit (Pierce, Thermo Fisher Scientific) was used to analyze the protein quantification of BEVs. First, BEVs are isolated and resuspended in PBS (Biosharp, Shanghai, China). Prepare a series of dilutions of the BEV samples to ensure that the protein concentration falls within the linear range of the assay. A common dilution factor is 1:10 to 1:100. Prepare a standard curve using a known concentration of bovine serum albumin (BSA) or other suitable protein standards provided with the kit. Create a series of dilutions (0, 10, 20, 40, 60, 80, 100 μg/mL). Add 25 μL of each standard and sample to separate wells of a microplate. Add 200 μL of BCA reagent (mixing equal parts of Reagent A and Reagent B) to each well. Incubate the plate at 37°C for 30 min. Measure the absorbance at 562 nm using a microplate reader (BioTek, Winooski, VT, USA). Construct a standard curve by plotting the absorbance values of the standards against their known concentrations. Calculate the protein concentration of the BEV samples by interpolating the absorbance readings on the standard curve. Finally, the protein‐to‐particles ratio (nearly 1 mg BEVs including 5 × 10^10^ particles) was determined to show the purity of vesicle suspensions.

### 2.6 In vitro assessment of HUVECs uptake efficiency of PKH67 green fluorescent dye‐labeled BEVs

Label BEVs with PKH67 green fluorescent dye (Thermo Fisher, USA) according to the manufacturer's instructions. Briefly, mix BEVs with PKH67 dye in a staining buffer, incubate for the recommended time, and then quench the reaction with BSA (Sigma Aldrich, USA). Dialyze the labeled BEVs to remove any unbound dye. Human umbilical vein endothelial cells (HUVECs) were purchased from the Cell Bank of the Chinese Academy of Sciences (Shanghai, China) and cultured with Ham's F‐12K, supplemented with 10% Fetal Bovine Serum (FBS, Gibco, USA), 0.1 mg/mL Heparin (Sigma Aldrich, USA), 0.05 mg/mL ECGS, (Corning, USA) and 1% Penicillin–Streptomycin (Gibco, USA). HUVECs at 10^6^ per dish (100 mm, Nest, Nanjing, China) were used for the assay. Add PKH67‐labeled BEVs to the HUVECs (50 μg/mL) and incubate for 6 h. After incubation, wash the cells thoroughly with PBS to remove any unbound BEVs. Fix the cells with a fixative such as 4% paraformaldehyde. Stain the cell nuclei with DAPI (4′,6‐diamidino‐2‐phenylindole) for visualization. Use a fluorescence microscope (CFX53, Olympus, Japan) to visualize the uptake of PKH67‐labeled BEVs by HUVECs.

### 2.7 CCK‐8 assay for cell viability

HUVECs at 10^7^ per dish (100 mm, Nest, Nanjing, China) were digested and adjusted to a cell concentration of 3 × 10^4^/mL. They were seeded into a 96‐well plate at 200 μL per well with 6 replicate wells per group. After cell adherence, each group was treated with ox‐LDL (100 μg/mL, Catalogue Number: 20605ES05, Yeasen Biotechnology (Shanghai) Co., Ltd) and BEVs (100 μg/mL). After 24 h of cell treatment, each well was supplemented with serum‐free culture medium containing 10% CCK‐8, followed by incubation at 37°C for 1 h. Absorbance was measured at 450 nm.

### 2.8 Transwell assay for cell migration

HUVECs at 10^7^ per dish (100 mm, Nest, Nanjing, China) were cultured in serum‐free medium for 12 h to eliminate serum effects. Cells were then digested, resuspended in serum‐free medium, counted, and adjusted to a cell concentration of 1 × 10^5^/mL. A volume of 200 μL of the cell suspension diluted with serum‐free medium was added to the upper chamber of a transwell insert, while 500 μL of medium containing 10% FBS was added to the lower chamber. After cell adherence, each group was treated with ox‐LDL (100 μg/mL, Catalogue Number: 20605ES05, Yeasen Biotechnology (Shanghai) Co., Ltd) and BEVs (100 μg/mL). After 24 h of cell treatment, the medium was removed, and the insert was washed three times with PBS, and then 800 μL of 4% paraformaldehyde was added to the lower chamber for fixation for 20 min. The insert was allowed to air dry, followed by staining with 1% crystal violet dye (Beyotime, Shanghai, China) for 10 min. The non‐migrated cells on the upper surface were gently wiped off with a cotton swab. After washing three times with PBS, the insert was air‐dried and placed under a cover glass on the objective of the microscope (CKX53, Olympus, Japan) for photography. Five random fields were selected for photography, and the cells that had migrated through the membrane were counted.

### 2.9 Cell apoptosis detection

HUVECs were plated at a density of 1 × 10^5^ cells/mL and seeded into a 6‐well plate, with 2 mL of cell suspension added to each well and three replicate wells per group. After allowing the cells to adhere, each group was treated with ox‐LDL (100 μg/mL, Catalogue Number: 20605ES05, Yeasen Biotechnology (Shanghai) Co., Ltd) and BEVs (100 μg/mL). Following 24 h of treatment, the cells were collected by centrifuging at 300*g* for 5 min at 4°C. Apoptosis was then detected using the FITC Annexin V Apoptosis Detection Kit with PI (BioLegend, USA). The cells were washed twice with pre‐chilled PBS, with centrifugation at 300*g* for 5 min at 4°C after each wash. A total of 1 × 10^5^ cells were collected, and the PBS was removed. The cells were then resuspended in 100 μL of 1 × binding buffer. Next, 5 μL of Annexin V‐FITC and 10 μL of PI staining solution were added, and the mixture was gently mixed. The cells were then incubated in the dark at room temperature for 10–15 min. After incubation, 400 μL of 1 × binding buffer was added, and the mixture was well mixed before placing on ice. The samples were analyzed within 1 h using a BD FACSLyric flow cytometer (BD Biosciences, USA). For flow cytometry analysis, a minimum of 10,000 events were acquired for each sample. The cell gating strategy involved selecting the cell population based on forward and side scatter properties, followed by analysis of Annexin V‐FITC and PI staining to quantify the percentage of apoptotic cells.

### 2.10 RNA transcriptome sequencing and GO and KEGG analysis

RNA was extracted and purified from cells using the RNeasy Mini Kit (Qiagen, Germany). The extracted RNA was then used for downstream applications. The extracted RNA was converted into cDNA libraries using the SuperScript IV First‐Strand Synthesis System (Invitrogen, USA) for cDNA synthesis, followed by library preparation using the NEBNext Ultra II DNA Library Prep Kit for Illumina (New England Biolabs, USA). The libraries were then subjected to high‐throughput sequencing on an Illumina NovaSeq 6000 platform (Illumina, USA) to generate millions of short sequence reads. The sequencing data underwent quality control using FastQC (Babraham Institute, UK) to assess the quality of the reads. Low‐quality and adapter sequences were removed using Trimmomatic (Usadel Lab, Germany), and potential contaminant sequences were filtered out using Kraken2 (Jensen Lab, USA) to improve data quality. The preprocessed sequencing data were aligned to the human reference genome (GRCh38) using HISAT2 (Johns Hopkins University, USA). The aligned reads were then quantified using FeatureCounts (Walter and Eliza Hall Institute of Medical Research, Australia) to determine the relative expression levels of each gene. Differential expression of genes was identified using the DESeq2 package (Bioconductor, USA) by comparing RNA expression levels between different samples. Statistical methods for differential expression analysis were applied to identify significantly differentially expressed genes. GO analysis was performed using the clusterProfiler package (Bioconductor, USA) with lists of differentially expressed genes. The genes were annotated to the three main domains (biological processes, molecular functions, and cellular components) to understand the enrichment of these genes in specific functions and processes. KEGG analysis was conducted using the clusterProfiler package (Bioconductor, USA) with lists of differentially expressed genes. The genes were mapped to specific metabolic pathways and biological functions to understand the enrichment of these genes in metabolic pathways and signaling pathways. The results of GO and KEGG analyses were interpreted, and visualization tools such as heatmaps, scatter plots, and pathway diagrams were generated using the ggplot2 package (R Project, USA) and pathview package (Bioconductor, USA) to explore patterns of change and potential functions in the RNA transcriptome.

### 2.11 In vivo immunofluorescence observation of the enrichment of PKH67‐labeled EC‐BEVs DHT in tissues

Label BEVs with PKH67 green fluorescent dye (Thermo Fisher, USA) as described above. Administer the PKH67‐labeled EC‐BEVs^DHT^ (2 mg/kg) to the male ApoE^−/−^ mice (Beijing Vital River Laboratory Animal Technology Co., Ltd.) via intravenous injection. Allow the animals to survive for 4 h to allow for biodistribution of the BEVs. Euthanize the animals and collect tissues of interest, including artery, brain, heart, and lung. Fix the tissues with a fixative such as 4% paraformaldehyde and process them for paraffin embedding. Mount the slides with a fluorescent mounting medium containing DAPI for nuclear staining. Use a fluorescence microscope (CFX53, Olympus, Japan) to visualize the enrichment of PKH67‐labeled EC‐BEVs^DHT^ in the tissues.

### 2.12 Mouse AS model

Eighteen male ApoE^−/−^ mice weighing 16–18 g, obtained from Beijing Vital River Laboratory Animal Technology Co., Ltd., were acclimated to standard chow for 7 days. They were then randomly divided into 3 groups with 6 mice each. Additionally, six male C57BL/6J ApoE^+/+^ mice weighing 16–18 g, obtained from Beijing Vital River Laboratory Animal Technology Co., Ltd., were acclimated to standard chow for 7 days and served as the blank control group. The control group mice had ad libitum access to standard chow, while the AS model group mice were fed a high‐fat diet for 8 weeks to induce the AS model. Meanwhile, the BEVs treatment group (2 mg/kg) received continuous tail vein injections for 8 weeks.

### 2.13 Mouse serum lipid levels and transaminase (ALT)/aspartate transaminase (AST)

After the final administration, mice were fasted but allowed free access to water for 12 h. Mice were then anesthetized via intraperitoneal injection of a 2% pentobarbital sodium solution (volume fraction), blood was collected from the orbital sinus, and serum was obtained after standing at room temperature for 30 min followed by centrifugation at 3000 rpm for 15 min. Serum levels of total cholesterol (TC), triglycerides (TG), low‐density lipoprotein cholesterol (LDL‐C), and high‐density lipoprotein cholesterol (HDL‐C) were measured using an automated biochemical analyzer. Serum ALT/AST levels were measured using Nanjing Kangcheng assay kits.

### 2.14 Mouse aortic oil red O staining

After blood collection from mice, cervical dislocation was performed, the chest cavity was opened, and the heart was exposed. PBS was rapidly perfused to remove residual blood, and the aorta (from the aortic arch to the abdominal aorta) was bluntly dissected, with surrounding adipose and connective tissues removed. The aorta was then fixed in 40 ng/L paraformaldehyde solution for 24 h, washed three times with distilled water, and placed in Oil Red O staining solution for 10–15 min. After differentiation in 75% ethanol, images were captured using an optical microscope to observe the distribution of atherosclerotic plaques in the aorta.

### 2.15 Hematoxylin and eosin (HE) staining

The aortic arch was taken and fixed with paraformaldehyde, dehydrated using a dehydrator, dehydrated with a series of gradient concentrations of ethanol, followed by alcohol benzene and xylene dehydration. The tissue was embedded in paraffin and cut into 4 μm sections. After deparaffinization, the sections were stained with hematoxylin staining solution, followed by bluing solution, and then counterstained with eosin staining solution. Finally, the sections were sealed with neutral resin. Pathological changes in the mouse aorta were observed under a microscope, images were captured, and analysis was performed.

### 2.16 Statistical analysis

Data were expressed as mean ± SD and analyzed by SPSS 24.0 package (SPSS Inc., Chicago, IL, USA). For comparisons between two groups, a t‐test was used. For comparisons among multiple groups, one‐way or two‐way ANOVA was used followed by a Tukey's post hoc test. Values less than 0.05 were considered statistically significant.

## 3 RESULTS

### 3.1 Construction, isolation, and characterization of ECs‐targeting and DHT‐loaded BEVs

**FIGURE 2:**
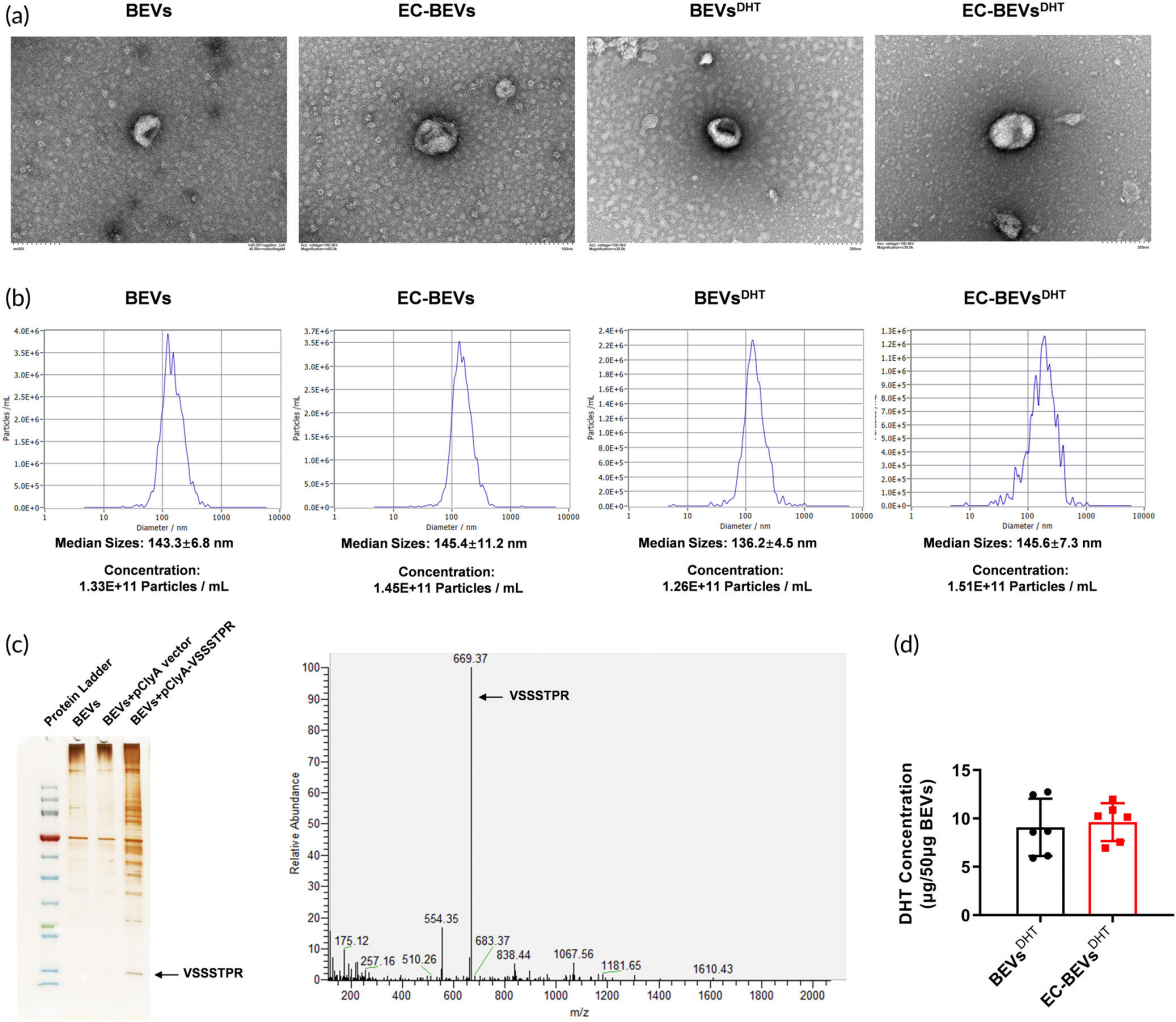
Characterization of ECs‐targeting and DHT loaded BEVs. (a) Morphology of BEVs by TEM; (b) Particle size and concentration analysis by NTA; (c) HPLC/LC–MS analysis verified the expression of ECs‐targeting peptide protein (VSSSTPR) expression in BEVs; (d) HPLC/LC–MS analysis of the content of DHT loaded in BEVs^DHT^ and EC‐BEVs^DHT^. *N* = 6, data = mean ± SD. Statistical differences between two groups were determined using a *t*‐test.

To enhance the bioavailability of DHT and tissue targeting while reducing toxic side effects, we inserted the coding sequence of ECs‐targeting peptides VSSSTPR into the pClyA plasmid and expressed it in LGG (Figure 1a). The clone 2 positive plasmid of pClyA‐hVec‐ECs (10xVSSSTPR) was selected by colony PCR (Figure 1b) and verified by restricted double digestion (Figure 1c). The overexpression of ClyA‐VSSSTPR in the recombinant strain was induced by IPTG. After multiple ultracentrifugation and ultrafiltration, bioengineered ECs‐targeting BEVs (EC‐BEVs) were obtained. DHT was then loaded into the BEVs and EC‐BEVs using electroporation techniques. Thus, we successively developed EC‐BEVs, DHT‐loaded BEVs (BEVs^DHT^), and DHT‐loaded ECs‐targeted BEVs (EC‐BEVs^DHT^). TEM and NTA indicated that all four types of BEVs from three independent batches exhibited typical spherical morphology (Figure 2a) with similar particle size distribution (BEVs: 143.3 nm, EC‐BEVs: 145.4 nm, BEVs^DHT^: 136.2 nm, EC‐BEVs^DHT^: 145.6 nm) and concentration (BEVs: 1.33E+11 Particles / mL, EC‐BEVs: 1.45E+11 Particles/mL, BEVs^DHT^: 1.26E+11 Particles/mL, EC‐BEVs^DHT^: 1.51E+11 Particles/mL) (Figure 2b). HPLC‐MS/MS confirmed the expression of the ECs‐targeting peptide VSSSTPR in EC‐BEVs^DHT^ (Figure 2c). Moreover, HPLC‐MS detected that the DHT content in BEVs^DHT^ and EC‐BEVs^DHT^ was 9.055 ± 1.839 μg/50 μg BEVs protein and 9.694 ± 1.473 μg/50 μg BEVs protein, respectively (Figure 2d).

### 3.2 EC‐BEVs^DHT^ attenuates ox‐LDL induced HUVEC injury

**FIGURE 3:**
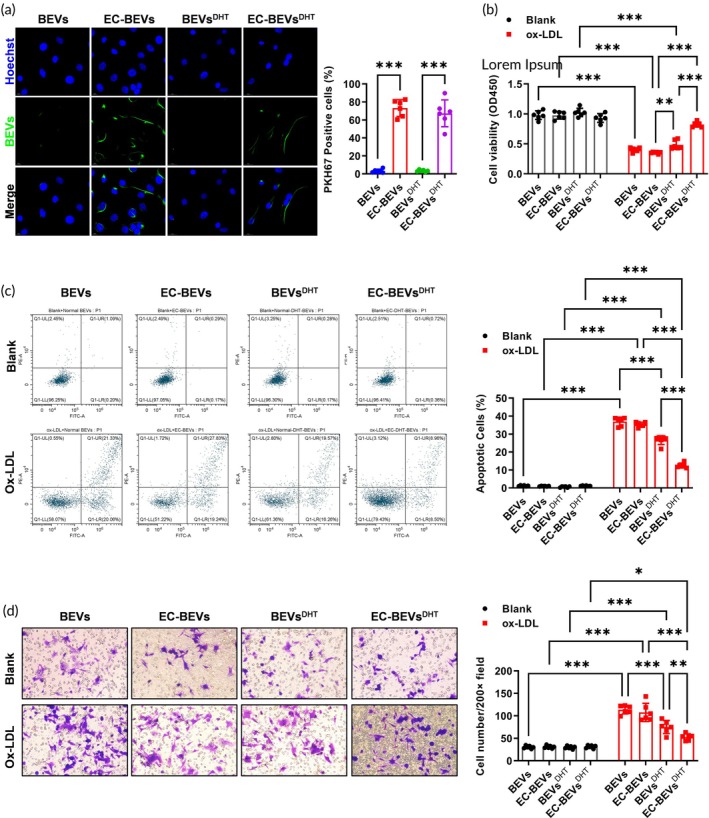
EC‐BEVs^DHT^ attenuates ox‐LDL induced HUVECs injury. (a) Assessment of HUVECs uptake efficiency of PKH67 green fluorescent dye‐labeled BEVs. (b) CCK‐8 assay was used to detect changes in cell viability before and after treatment with ox‐LDL and BEVs. (c) Flow cytometry using Annexin V/PI staining to evaluate changes in cell apoptosis levels before and after treatment with ox‐LDL and BEVs. (d) Transwell assay was performed to measure changes in cell migration ability before and after treatment with ox‐LDL and BEVs. *N* = 6, data = mean ± SD, *p <0.05, **p <0.01, ***p <0.001, compared with indicated groups. Statistical differences between groups were determined using Two‐way ANOVA followed by a Tukey's post hoc test.

After labeling with PKH67 fluorescence, BEVs, EC‐BEVs, BEVs^DHT^, and EC‐BEVs^DHT^ at a concentration of 100 μg/mL were co‐incubated with HUVECs. It was found that EC‐BEVs and EC‐BEVs^DHT^ exhibited higher affinity toward HUVECs (Figure 3a). A foam cell model was established by stimulating HUVECs with 100 μg/mL ox‐LDL. Subsequently, BEVs, EC‐BEVs, BEVs^DHT^, and EC‐BEVs^DHT^ at the same concentration were separately added. Notably, 100 μg/mL ox‐LDL incubation for 24 h led to a significant decrease in cell viability. Pretreatment with BEVs, EC‐BEVs, BEVs^DHT^, and EC‐BEVs^DHT^ had no significant influence on cell viability (Figure 3b). However, it was observed that EC‐BEVs^DHT^ more effectively attenuated the ox‐LDL‐impaired cell viability of HUVECs (Figure 3b). We also examined the effect of BEVs, EC‐BEVs, BEVs^DHT^, and EC‐BEVs^DHT^ on apoptosis. Moreover, flow cytometry results revealed that the 100 μg/mL ox‐LDL‐induced HUVEC apoptosis (% annexin V‐positive plus % PI‐positive and negative cells) was dramatically reversed by EC‐BEVs^DHT^(Figure 3c). For the migration morphology, 100 μg/mL ox‐LDL significantly increased the migration ability of HUVECs, which was suppressed by EC‐BEVs^DHT^ treatment (Figure 3d). These results demonstrated that EC‐BEVs^DHT^ attenuates ox‐LDL‐induced HUVEC injury in vitro.

### 3.3 In vivo distribution and cytotoxicity of EC‐BEVs^DHT^

**FIGURE 4:**
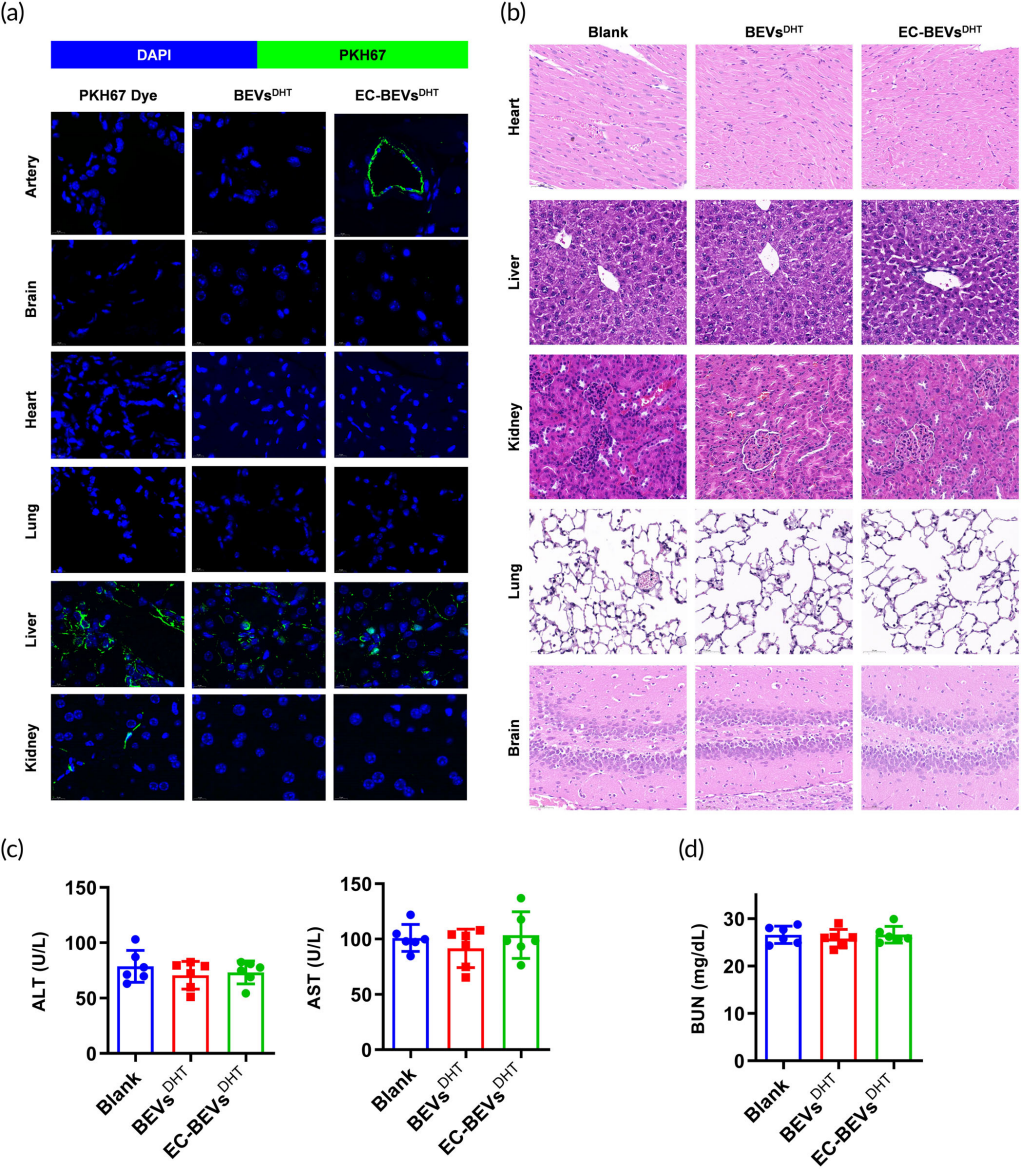
In vivo distribution and cytotoxicity of EC‐BEVs^DHT^. (a) Immunofluorescence observation of the enrichment of PKH67‐labeled EC‐BEVs^DHT^ in tissues including artery, brain, heart, and lung; (b) HE staining of multiple organs indicating no significant toxicity of EC‐BEVs^DHT^ to tissues including heart, liver, kidney, lung, and brain; (c) ALT, AST, and (d) blood urea nitrogen values indicating no significant liver or kidney toxicity of BEVs^DHT^ and EC‐BEVs^DHT^. *N* = 6, data = mean ± SD. Statistical differences between groups were determined using one‐way ANOVA followed by a Tukey's post hoc test.

Targeted therapy is one of the main strategies to enhance the efficiency and reduce side effects of agents. We, therefore, evaluated the in vivo distribution of EC‐BEVs^DHT^. We applied PKH67 fluorescent dye to label BEVs^DHT^ and EC‐BEVs^DHT^. Eight‐week‐old female C57BL/6 mice were randomly separated into two groups: BEVs^DHT^ and EC‐BEVs^DHT^. The fluorescence intensity of the artery, brain, heart, lung, liver, and kidney in each group was measured 4 h after tail vein injection of PKH67 dye or BEVs (Figure 4a). Compared with other groups, a specific dye was enriched in the artery of the EC‐BEVs^DHT^ injection mice, which indicated that EC‐BEVs^DHT^ specifically targeted the artery. The in vivo long‐term cytotoxicity of BEVs^DHT^ and EC‐BEVs^DHT^ was then assessed after intravenous administration. As shown in Figure 4b, no distinct pathological changes were observed in the heart, liver, kidney, lung, and brain after 4 weeks of administration. In addition, after 24 h of administration, the liver and kidney‐related biochemical indicators such as ALT (Figure 4c), AST (Figure 4c), and blood urea nitrogen (BUN, Figure 4d) were also evaluated. There was no significant difference in liver and renal function, indicating that the EC‐BEVs^DHT^ have no short‐term cytotoxicity. Collectively, we successfully constructed ECs‐targeting bioengineered BEVs as novel DHT‐loaded nanocarriers without long‐term or short‐term in vivo toxicity.

### 3.4 EC‐BEVs^DHT^ decreases AS in ApoE^−/−^ mice

**FIGURE 5:**
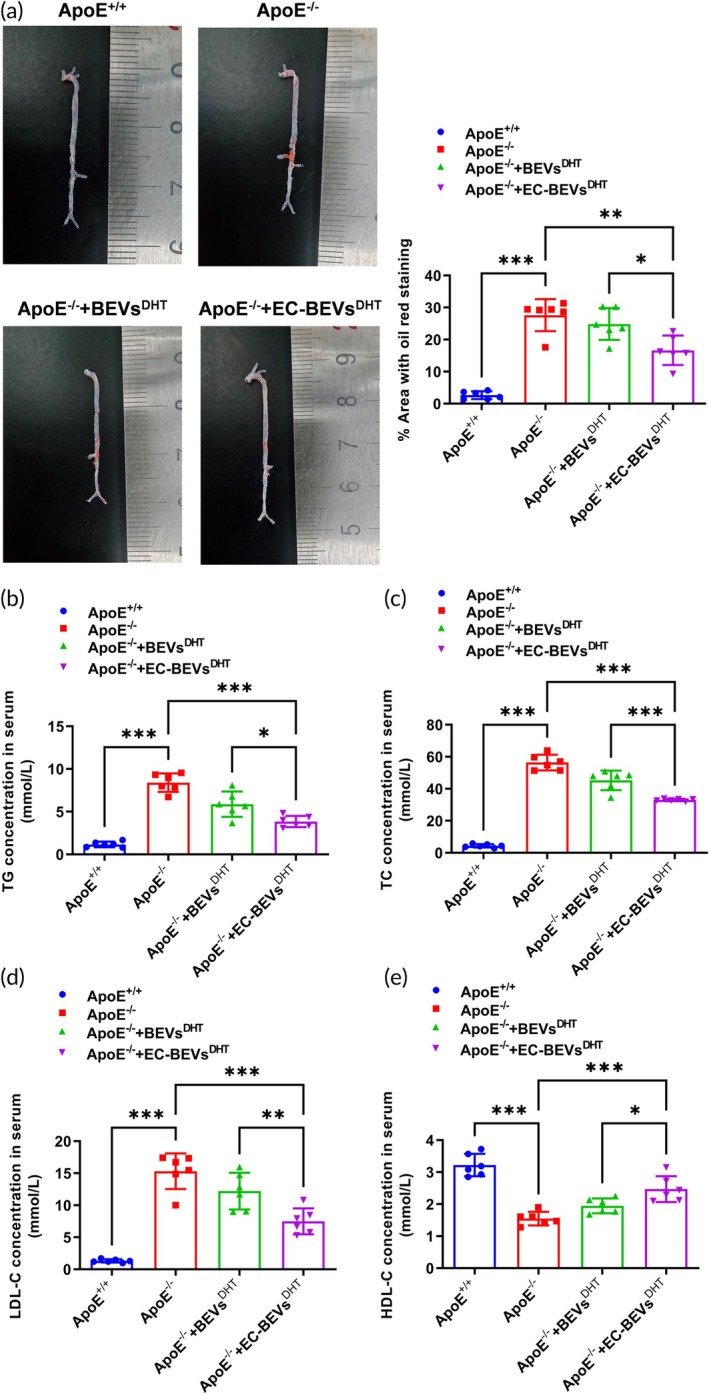
EC‐BEVs^DHT^ decreases AS in ApoE^−/−^ mice. (a) Oil red O staining of the aorta revealing a significant reduction in atherosclerotic plaques in ApoE^−/−^ mice treated with EC‐BEVs^DHT^; (b). EC‐BEVs^DHT^ markedly decreased serum levels of TG (B) and TC (c), as well as LDL‐C (d) in ApoE^−/−^ mice; (e) EC‐BEVs^DHT^ significantly increased serum levels of HDL‐C in ApoE^−/−^ mice. N = 6, data = mean ± SD, *p <0.05, **p <0.01, ***p <0.001, compared with indicated groups. Statistical differences between groups were determined using One‐way ANOVA followed by a Tukey's post hoc test.

**FIGURE 6:**
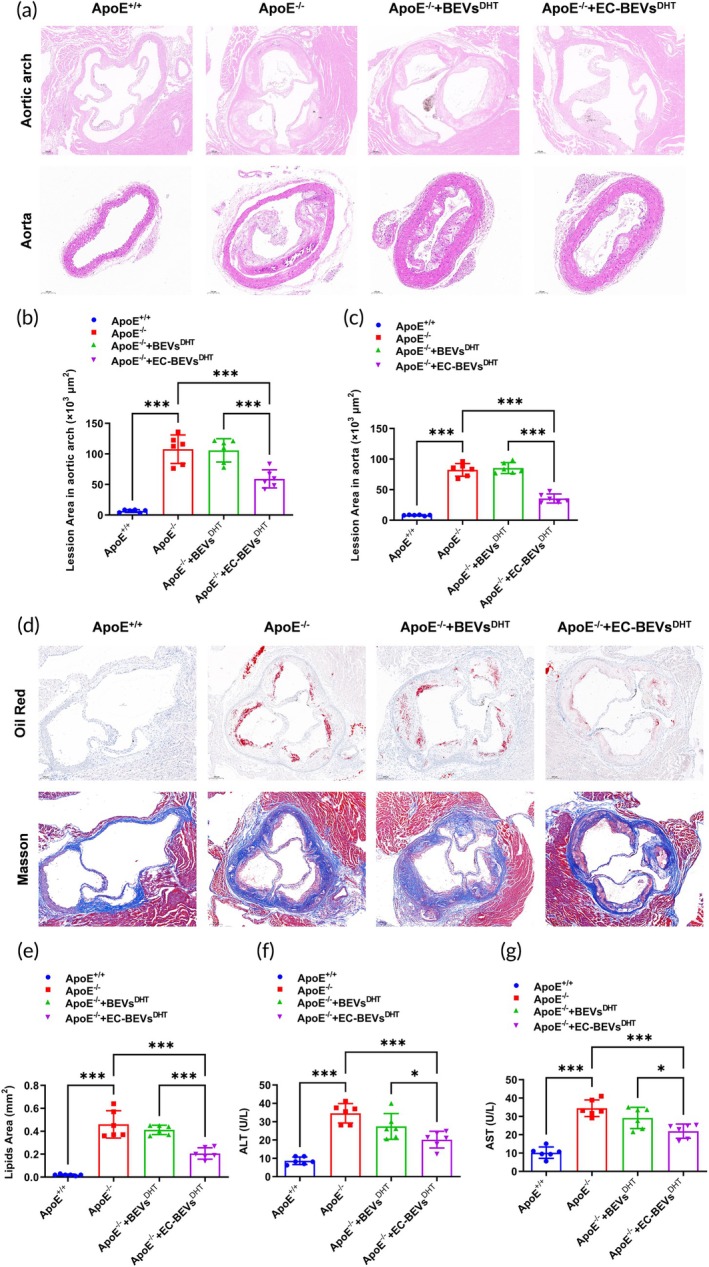
EC‐BEVs^DHT^ decreases atherosclerotic plaques and lipid accumulation in ApoE^−/−^ mice. (a)–(c) HE staining revealed a significant reduction in the number of aortic arch and aortic plaque areas in ApoE^−/−^ mice treated with EC‐BEVs^DHT^. (d), (e) Oil red O staining and masson trichrome staining showed a significant decrease in lipid content and fibrosis degree in the aortic arch of ApoE^−/−^ mice treated with EC‐BEVs^DHT^; (f), (g) EC‐BEVs^DHT^ markedly reduced the activity of serum ALT and AST in ApoE^−/−^ mice. *N* = 6, data = mean ± SD, *p <0.05, **p <0.01, ***p <0.001, compared with indicated groups. Statistical differences between groups were determined using One‐way ANOVA followed by a Tukey's post hoc test.

AS was quantified in ApoE^−/−^ mice and control ApoE^+/+^ littermates. Oil red staining was performed to evaluate the severity of aortic AS. The results of gross morphology in aortic arches showed that the ApoE^−/−^ mice reported more lipid accumulation in arterial plaques than that of the control ApoE^+/+^ group (Figure 5a). However, the EC‐BEVs^DHT^ treatment significantly lowered the lipid accumulation when compared with the normal ApoE^−/−^ mice and BEVs^DHT^ treated ApoE^−/−^ mice groups (Figure 5a). We next examined the circulating lipid profile of TG, T‐CHO, LDL‐C, and HDL‐C, and found that TG (Figure 5b), T‐CHO (Figure 5c), and LDL‐C (Figure 5d) were significantly increased and HDL‐C decreased in ApoE^−/−^ mice, among which EC‐BEVs^DHT^ treatment significantly attenuated the increased TG (Figure 5b), T‐CHO (Figure 5c), and LDL‐C (Figure 5d), and increased the HDL‐C in ApoE^−/−^ mice. Furthermore, HE staining was performed to evaluate the severity of aortic AS in ApoE^−/−^ mice and control ApoE^+/+^ littermates, which showed no obvious atherosclerotic plaques in the ApoE^+/+^ mice but obvious atherosclerotic plaques in the aortic arch and aorta from ApoE^−/−^ mice (Figure 6a), featured with obvious atherosclerotic plaques on the surface of intima and increased lesion areas in aortic arch (Figure 6b) and aorta (Figure 6c). Compared with the normal ApoE^−/−^ mice and BEVs^DHT^ treated ApoE^−/−^ mice groups, EC‐BEVs^DHT^ treated mice showed only local bulging plaques on the intimal surface with complete fiber caps at the top of the plaques and significant decreases of lesion areas in aortic arch (Figure 6b) and aorta (Figure 6c). Oil red staining was further used to evaluate the severity of aortic AS, which showed that the ApoE^−/−^ mice group reported more lipid accumulation in arterial plaques than control ApoE^+/+^ littermates (Figure 6d). However, EC‐BEVs^DHT^ treatment significantly lowered the lipid accumulation (Figure 6d). We also characterized the collagen composition of aortic root by Masson trichrome staining of lesions with subsequent quantification. Consistently, a significant increase of collagen content from ApoE^−/−^ mice were observed and EC‐BEVs^DHT^ treatment significantly lowered collagen content (Figure 6d). To evaluate whether there was any potential liver protective effect of EC‐BEVs^DHT^, we measured plasma ALT (Figure 6e) and AST (Figure 6f). These hepatic enzymes were obviously increased in ApoE^−/−^ mice compared with the control ApoE^+/+^ littermates. Interestingly, EC‐BEVs^DHT^ significantly attenuated these increases in ApoE^−/−^ mice. In conclusion, these results suggested that EC‐BEVs^DHT^ decreases AS in ApoE^−/−^ mice.

### 3.5 Effect of EC‐BEVs^DHT^ in the transcriptome of HUVECs and ApoE^−/−^ mice aorta tissues

**FIGURE 7:**
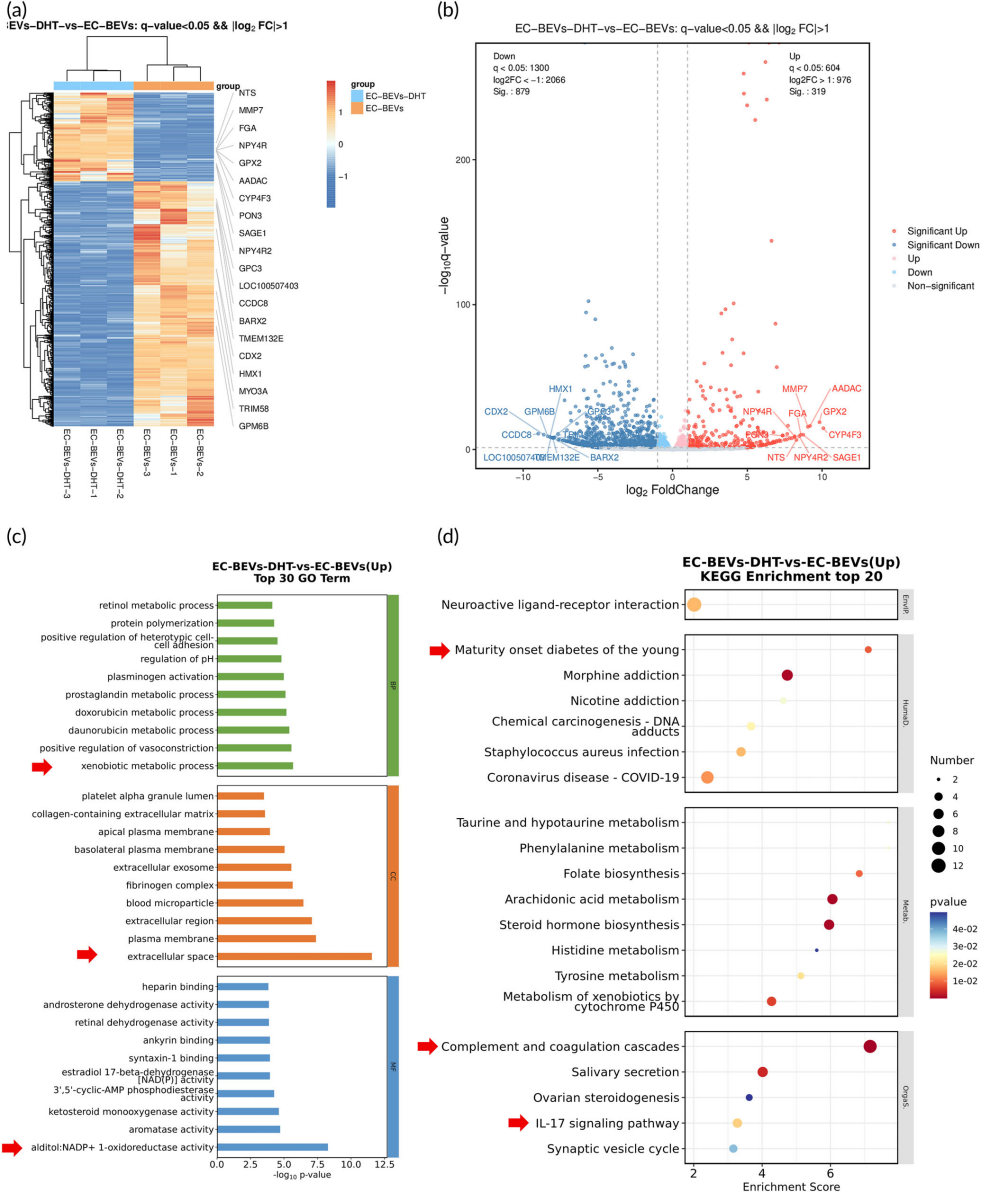
Effect of EC‐BEVs^DHT^ in the transcriptome of HUVECs. (a), (b) Heatmap and volcano map displaying expression levels of differentially up‐regulated related genes; (c) GO analysis of functionally relevant genes, indicating significant activation in genes related to xenobiotic metabolic processes, extracellular space, and NADP+ 1‐oxidoreductase activity; (d) KEGG analysis of differential signaling pathways in response to up‐regulated genes, suggesting activated signaling including maturity onset diabetes of the young, complement and coagulation cascades, and IL‐17 signaling pathway.

**FIGURE 8:**
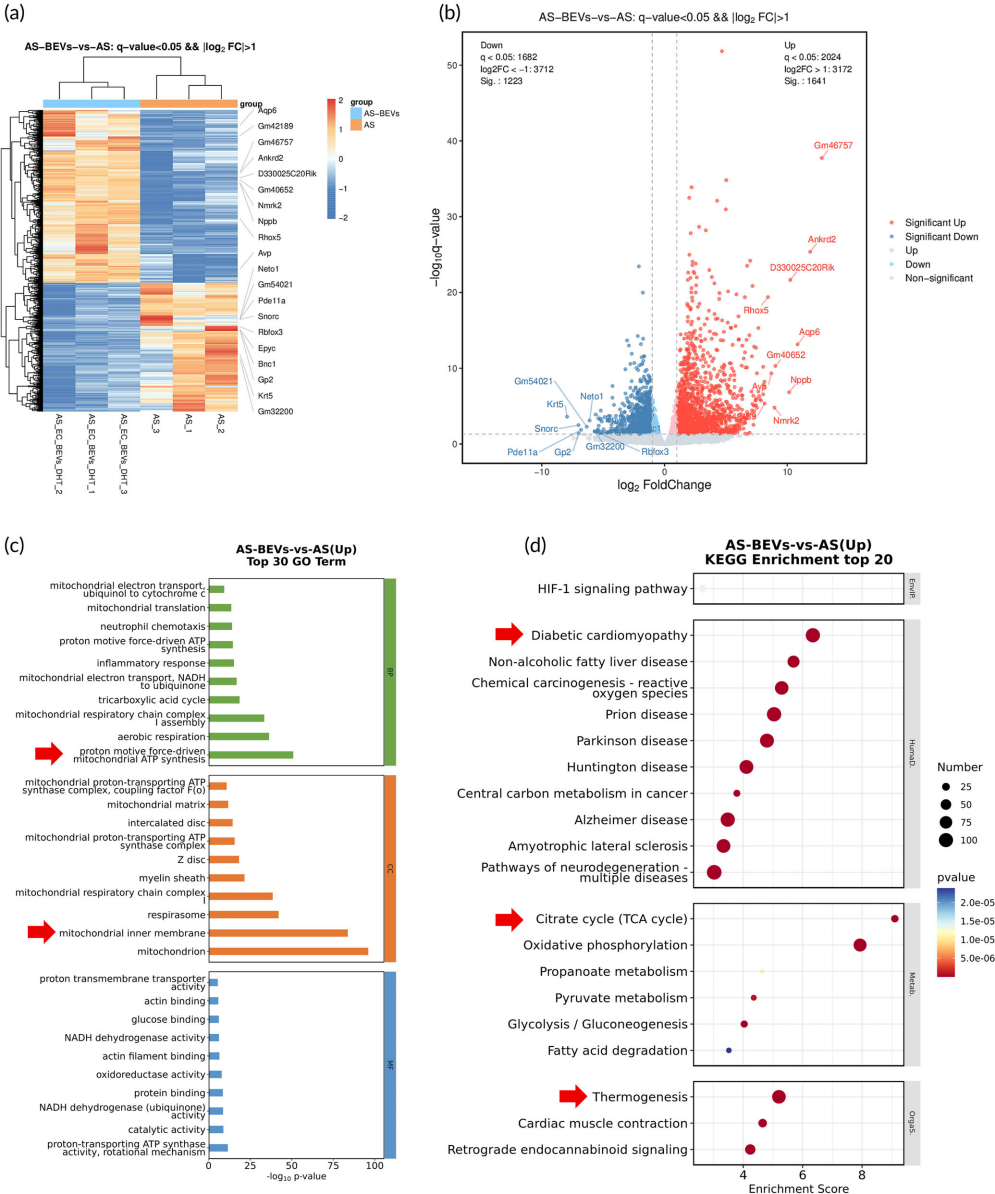
Effect of EC‐BEVs^DHT^ in the transcriptome of ApoE^−/−^ mice aorta tissues. (a), (b) Heatmap and volcano displaying expression levels of differentially related genes; (b) GO analysis of functionally up‐regulated relevant genes, indicating significant activation in genes related to proton motive force‐derived mitochondrial ATP synthesis and mitochondrion; (c) KEGG analysis of differential signaling pathways response to up‐regulated genes, suggesting activated signaling including diabetic cardiomyopathy, citrate cycle, and thermogenesis.

RNA‐seq results revealed thousands of genes altered by EC‐BEVs^DHT^ in HUVECs. The Heatmap and volcano map of differently expressed genes (DEGs) among different treatment groups are reported in Figure 7a,b. Compared to control HUVECs, 368 DEGs were identified in EC‐BEVs treated HUVECs. Meanwhile, 2648 DEGs were identified in EC‐BEVs^DHT^ treated HUVECs. The DEG‐based GO analysis and KEGG pathway enrichment results in response to the up‐regulated genes were reported in Figure 7c,d. Major pathways observed with remarkable changes included maturity onset diabetes of the young, complement and coagulation cascades, and IL‐17 signaling pathway. Consistently, RNA‐seq results revealed thousands of genes altered by EC‐BEVs^DHT^ in ApoE^−/−^ mice aorta tissues. The Heatmap and volcano map of DEGs among different treatment groups are reported in Figure 8a,b. Compared to control ApoE^+/+^, 1358 DEGs were identified in ApoE^−/−^ mice aorta tissues. Compared with ApoE^−/−^ mice aorta tissues, 692 DEGs were identified in EC‐BEVs^DHT^ treated ApoE^−/−^ mice aorta tissues. The DEG‐based GO analysis and KEGG pathway enrichment results in response to the up‐regulated genes results were reported in Figure 8c,d. Major pathways observed with remarkable changes included diabetic cardiomyopathy, citrate cycle, and thermogenesis signaling pathways.

## 4 DISCUSSION

**FIGURE 9:**
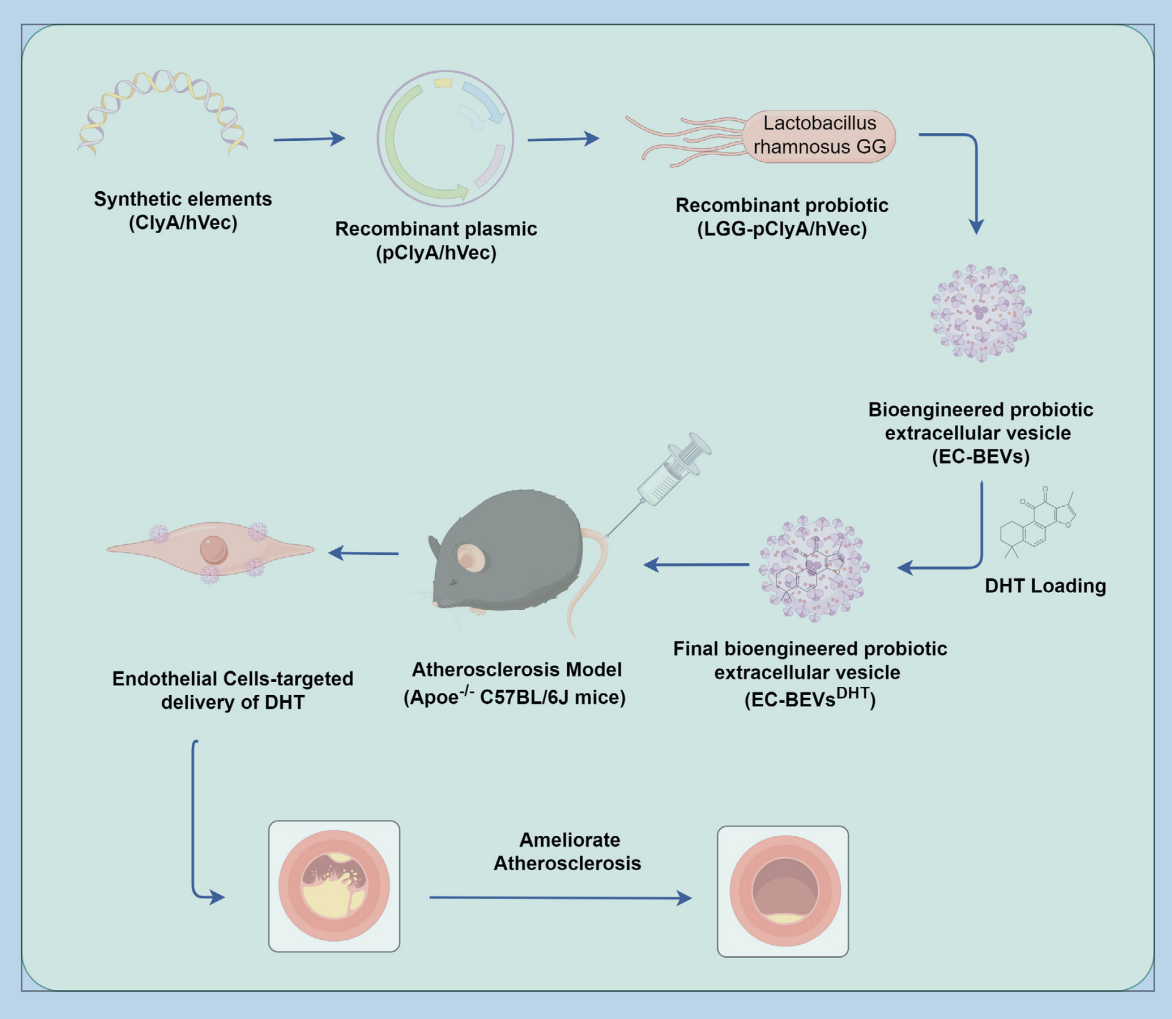
Schematic diagram of constructing ECs‐targeted BEVs loaded with DHT and its therapeutic strategy for AS.

DHT can alleviate AS, but its bioavailability and tissue targeting need to be improved.^7^, ^11^, ^12^ Nanoscale drug delivery carrier BEVs with stable drug loading capacity, good biocompatibility, easy modification, and large‐scale production potential are expected to address this challenge.^15^, ^21^, ^22^, ^23^ In this study, we designed and constructed EC‐BEVs^DHT^ using the probiotic LGG and ECs‐targeting peptide, and evaluated anti‐AS effects in HUVECs and ApoE^−/−^ mice (Figure 9).

As one of the main active components of Salvia miltiorrhiza, DHT has been shown to significantly inhibit inflammation, oxidative stress, and interstitial calcification.^24^, ^25^ Previous studies have found that DHT can inhibit ox‐LDL uptake and monocyte adhesion, reduce blood lipids, and decrease and stabilize AS plaques.^26^, ^27^, ^28^, ^29^ Although DHT has significant clinical value, its small molecular weight, short half‐life, and poor water solubility limit its bioavailability and tissue targeting. BEVs produced by LGG possess unique characteristics such as a cell‐free system, nanometer‐scale structure, stable drug‐loading capacity, good biocompatibility, ease of modification, and scalability for mass production.^17^, ^30^, ^31^, ^32^, ^33^, ^34^ Therefore, based on the principles of materials synthesis biology, we designed and constructed EC‐BEVs^DHT^ using the probiotic LGG and ECs‐targeting peptide. By inserting the coding sequence of ECs‐targeting peptides (VSSSTPR)^35^ into the pClyA plasmid and expressing it in LGG, we successfully engineered BEVs with specific affinity for ECs. The subsequent loading of DHT into these BEVs using electroporation techniques further expanded the versatility of this system, enabling targeted delivery of the therapeutic compound. TEM and NTA revealed that all four types of BEVs maintained typical spherical morphology and exhibited uniform particle size distribution, indicative of their stability and homogeneity.^17^ The successful expression of the ECs‐targeting peptide VSSSTPR in EC‐BEVs^DHT^ confirmed the functionalization of the vesicles with the targeting ligand. This is a significant achievement as it validates the specific affinity of the engineered BEVs toward ECs, potentially improving the precision and efficiency of drug delivery to the target site.^35^ Moreover, the quantification of DHT content in BEVs^DHT^ and EC‐BEVs^DHT^ using HPLC‐MS demonstrated efficient loading of the therapeutic compound into the vesicles. The preferable drug release efficiency observed at different time points indicates sustained and controlled release of DHT from both BEVs^DHT^ and EC‐BEVs^DHT^ formulations. The engineered BEVs exhibit promising properties including specific targeting ability toward ECs, efficient encapsulation of DHT, and sustained release kinetics. These findings lay the foundation for further development and optimization of BEVs‐based drug delivery systems for various therapeutic applications, particularly in the treatment of ECs‐related diseases.

The ox‐LDL plays a critical role in the pathogenesis of AS, a chronic inflammatory disease characterized by the accumulation of lipids and fibrous elements in arterial walls.^36^, ^37^ HUVECs are commonly used in vitro models to study AS due to their relevance to endothelial dysfunction, an early event in AS.^38^, ^39^ The ox‐LDL reflects the oxidative stress conditions present in atherosclerotic lesions, making it a clinically relevant inducer of cellular damage.^40^, ^41^ When exposed to ox‐LDL, HUVECs exhibit impaired endothelial function, characterized by decreased nitric oxide production, increased expression of adhesion molecules, and enhanced permeability, mirroring early events in AS.^42^ The response of HUVECs to ox‐LDL, including inflammation, apoptosis, and endothelial‐to‐mesenchymal transition, recapitulates key aspects of AS, providing insights into disease mechanisms and potential therapeutic targets.^43^ By studying the effects of ox‐LDL on HUVECs over time, it can simulate different stages of AS progression, from endothelial dysfunction to plaque formation, facilitating the development and testing of novel anti‐atherosclerotic therapies.^44^, ^45^ The in vitro findings presented in this study highlight the potential of EC‐BEVs^DHT^ in attenuating ox‐LDL‐induced HUVECs injury. We firstly demonstrated the enhanced affinity of EC‐BEVs and EC‐BEVs^DHT^ toward HUVECs, indicating successful modification with EC‐targeting peptides. Importantly, EC‐BEVs^DHT^ exhibited superior protective effects against ox‐LDL‐induced HUVECs injury compared to other formulations, as evidenced by improved cell viability and reduced apoptosis. Furthermore, the observed suppression of ox‐LDL‐induced HUVECs migration by EC‐BEVs^DHT^ underscores the potential role of this formulation in preventing the progression of AS, a condition associated with aberrant EC function and migration. These results not only validate the therapeutic potential of EC‐BEVs^DHT^ but also shed light on the underlying mechanisms by which they exert their protective effects, potentially paving the way for the development of novel therapeutic strategies for EC‐related disorders.

The ApoE^−/−^ mouse model has been extensively utilized in AS research due to its propensity to develop atherosclerotic lesions similar to those observed in humans.^46^ This model offers valuable insights into the cellular and molecular mechanisms underlying AS and serves as a crucial tool for evaluating AS cell models.^47^ It lacks functional ApoE^−/−^ mice, resulting in impaired clearance of plasma lipoproteins and an accumulation of cholesterol‐rich particles, leading to the development of atherosclerotic lesions.^48^ This genetic predisposition closely resembles the dyslipidemia observed in humans with AS.^49^ The ApoE^−/−^ mice also develop atherosclerotic lesions in a predictable and reproducible manner, allowing researchers to study lesion morphology, composition, and progression over time.^50^ This enables the evaluation of cellular responses within the lesions, including inflammation, foam cell formation, and smooth muscle cell proliferation. Thus, the ApoE^−/−^ mouse AS model plays a crucial role in evaluating AS cell models by providing a relevant and translational platform for studying disease pathogenesis, testing therapeutic interventions, and understanding the cellular and molecular mechanisms involved in lesion development and progression.^51^ The in vivo ApoE^−/−^ AS model results presented in this study demonstrate the promising potential of EC‐BEVs^DHT^ in mitigating AS in ApoE^−/−^ mice. We showed compelling evidence of the therapeutic efficacy of EC‐BEVs^DHT^ in reducing aortic lipid accumulation, improving lipid profile, and attenuating atherosclerotic plaque formation. Oil red staining revealed a significant reduction in lipid accumulation in the aortic plaques of mice treated with EC‐BEVs^DHT^ compared to untreated ApoE^−/−^ mice and those treated with BEVs^DHT^ alone, indicative of the potent anti‐atherogenic effects of the therapeutic formulation. Moreover, histological analysis demonstrated a marked decrease in atherosclerotic lesion areas in both the aortic arch and aorta of EC‐BEVs^DHT^‐treated mice, accompanied by the presence of local bulging plaques with complete fibrous caps, suggesting stabilization of the plaques. Furthermore, EC‐BEVs^DHT^ treatment was associated with a favorable modulation of the circulating lipid profile, characterized by a significant decrease in TC, TG, and LDL cholesterol, along with an increase in HDL cholesterol levels, indicating improved lipid metabolism and athero protective effects. Additionally, Masson trichrome staining revealed a reduction in collagen content within the aortic lesions of EC‐BEVs^DHT^‐treated mice, suggesting a potential role in plaque remodeling and fibrous cap stabilization. These findings collectively underscore the robust anti‐atherosclerotic properties of EC‐BEVs^DHT^ and suggest their potential as a promising therapeutic strategy for the management of atherosclerotic cardiovascular disease.

The transcriptomic analysis of HUVECs and ApoE^−/−^ mice aorta tissues following treatment with EC‐BEVs^DHT^ revealed significant alterations in gene expression profiles, indicative of the underlying molecular mechanisms mediating the protective effects of this therapeutic formulation. In both cell and tissue models, treatment with EC‐BEVs^DHT^ resulted in substantial changes in gene expression patterns, particularly affecting pathways associated with inflammatory responses, such as TNF signaling, NF‐κB activation, cytokine‐cytokine receptor interaction, and ECM‐receptor interaction. Additionally, pathways related to cell survival and signaling cascades, including JAK–STAT and PI3K‐AKT pathways, were also modulated by EC‐BEVs^DHT^ treatment. These findings suggest a multifaceted mechanism of action underlying the anti‐atherosclerotic effects of EC‐BEVs^DHT^, involving the attenuation of inflammatory processes and the promotion of cell survival and signaling pathways. Further elucidation of these molecular pathways may provide valuable insights into the development of targeted therapeutic interventions for AS and other ECs‐related disorders.

In conclusion, our findings suggest that EC‐BEVs^DHT^ hold promise as a safe and effective therapeutic strategy for AS, offering potential advantages over traditional treatments.

## AUTHOR CONTRIBUTIONS

Zhong‐yong Liu, Rong‐rong Zhu, and Xue‐liang Zhou designed this study; Rong‐rong Zhu and Xue‐liang Zhou performed all experiments; Rong‐rong Zhu, Xue‐liang Zhou, Yan‐wei Liu, Ri Xu, and Peng Deng analyzed and interpreted the data; Rong‐rong Zhu and Zhong‐yong Liu drafted the manuscript. All authors read and approved the final manuscript.

## FUNDING INFORMATION

This work was supported by grants from the National Natural Science Foundation of China (82360060, 82260918), NATCM's Project of High‐level Construction of Key TCM Disciplines (ZYYZDXK‐2023113), National Famous Traditional Chinese Medicine Expert Inheritance Studio Construction Project (2022‐75), the Natural Science Foundation of Jiangxi Province (20232ACB206003, 20232BAB206146,20243BCE51022).

## CONFLICT OF INTEREST STATEMENT

The authors declare no conflict of interest.

## Data Availability

The data that support the findings of this study are available from the corresponding author upon reasonable request.
